# A Case of Complete and Durable Molecular Remission of Chronic Lymphocytic Leukemia Following Treatment with Epigallocatechin-3-gallate, an Extract of Green Tea

**DOI:** 10.7759/cureus.441

**Published:** 2015-12-29

**Authors:** Dawn Lemanne, Keith I Block, Bruce R Kressel, Vikas P Sukhatme, Jeffrey D White

**Affiliations:** 1 Oncology, Oregon Integrative Oncology; 2 Block Center for Integrative Cancer Treatment, Department of Medicinal Chemistry and Pharmacognosy, College of Pharmacy, University of Illinois at Chicago; 3 Medical Oncology/Hematology, The Johns Hopkins University School of Medicine; 4 School of Medicine, BIDMC, Harvard Medical School; 5 Director, Office of Cancer Complementary and Alternative Medicine, National Cancer Institute, US National Institutes of Health

**Keywords:** chronic lymphocytic leukemia, spontaneous remission, egcg, curcumin, secondary autoimmune hemolytic anemia, green tea, camilla sinensis, epigallocatechin, integrative oncology

## Abstract

We report the case of a 48-year-old man who achieved a complete molecular remission 20 years after a diagnosis of chronic lymphocytic leukemia while using epigallicatechin-3-gallate, an extract of green tea. The patient presented at age 28 with lymphocytosis, mild anemia, mild thrombocytopenia, and massive splenomegaly, for which a splenectomy was performed. He was then followed expectantly. Over the next two decades, he suffered two symptomatic chronic lymphocytic leukemia-related events. The first occurred twelve years after diagnosis (at age 40) when the patient developed fevers, night sweats, and moderate anemia. He was diagnosed with autoimmune hemolytic anemia secondary to chronic lymphocytic leukemia. The patient declined conventional therapy in favor of a diet, exercise, and supplement regimen, and recovered from the autoimmune hemolytic anemia though the underlying chronic lymphocytic leukemia remained evident. This is the first published case report of "spontaneous" recovery from secondary autoimmune hemolytic anemia in an adult.

Over the second decade following chronic lymphocytic leukemia diagnosis, serial bone marrow biopsies demonstrated increasing lymphocytosis, with minimal peripheral lymphocytosis. However, twenty years after diagnosis, peripheral lymphocytosis accelerated, with white blood cell counts rising to 55,000/µL. Because the patient continued to refuse conventional therapy, he was treated instead with a supplement regimen that included high doses of epigallocatechin-3-gallate, a green tea extract. Peripheral lymphocytosis resolved. More remarkably, a bone marrow examination, including flow cytometry, showed no evidence of a malignant clone. Two years later (at age 51), the peripheral blood and bone marrow were without molecular evidence of chronic lymphocytic leukemia or any malignancy. The patient remains well at age 52.

## Introduction

Clinical remission of chronic lymphocytic leukemia without conventional therapy is a rare event, estimated to occur at the rate of 1% per year among cases of CLL [1]. Moreover, most such remissions are incomplete; a recent review of 21 such cases found persistent monoclonal B lymphocytosis in 67%, with only 33% regressing to a molecularly normal phenotype. In addition, the more time elapsed between diagnosis and remission, the greater the likelihood of persistent monoclonal B lymphocytosis [1]. Here, however, we describe a case of complete clinical and molecular regression of chronic lymphocytic leukemia achieved without conventional systemic treatment and occurring twenty years after diagnosis.

## Case presentation

An asymptomatic 28-year-old man presented in September 1991 with massive splenomegaly, mild anemia, and thrombocytopenia. Hemoglobin was 12.5 g/dL, platelet count was 105,000/µL, and total white blood cell count measured 5700/µL, with 61.3% lymphocytes. Peripheral and central lymphadenopathy were absent.

Peripheral blood smear showed atypical lymphocytes, a few with cytoplasmic projections. Polymerase chain reaction (PCR) and flow cytometry studies demonstrated a monoclonal B-cell population expressing CD19 and CD20. There was only partial co-expression of CD5 and CD11c. Bone marrow was normocellular, but with multiple atypical lymphoid aggregates and decreased iron stores.

Elective splenectomy was performed in November 1991. The splenic specimen contained diffuse infiltrates of small round lymphocytes with round to oval nuclei, consistent with chronic lymphocytic leukemia/small lymphocytic lymphoma.

From 1992 to 2003, the patient remained asymptomatic, but serial bone marrow biopsies revealed increasing lymphocytic infiltration (Table 1). During this period, the peripheral white blood cell count fluctuated between 5.3 and 14.8 g/dL. In 2001, loss of CD5 expression was noted.

**Table 1:** Bone marrow examination findings, 1991-2014 Abbreviations: BM = bone marrow; PCR = polymerase chain reaction; GWU = George Washington University, NIH = National Institutes of Health

Year	BM Cellularity	BM Lymphocytic Infiltration	PCR Findings	Flow Cytometry	Reviewer Comments	Reviewing Institution
1991	50%	10%	Monoclonal lymphocytes, CDR3+	CD5 “partial co-expression” with CD11c (peripheral blood)	Normocellular marrow; low grade lymphoprolferative disorder of B cell origin	GWU, NIH Harvard
1992	40%	“3 small aggregates of small round lymphocytes”			Normocellular; CLL or small lymphocytic lymphoma	GWU
1993 (Mar)	40%	5-10%			CLL; no significant interval change	GWU
1993 (Sept)	50%	10%				GWU
1994	50%	10-15%				GWU
1997	40%	30%				Johns Hopkins
1998	50%	10-20%				Johns Hopkins
2000	60%	70%				GWU
2001	80%	50%		CD5 not expressed		GWU, Harvard, Johns Hopkins
2002	90-100%	80%		CD5 not expressed		GWU, Harvard
2003	90-100%	“Almost replaced”			Marrow replaced by diffuse lymphocytic infiltrate	GWU, Johns Hopkins
2006	60-70%	30%		CD5 not expressed	Evidence of CLL; percentage of involvement by lymphocytes slightly less pronounced	GWU
2012	60%		IgHV rearrangement not seen		“Diagnostic features of a lymphoproliferative disorder are not seen”	Harvard
2014	80%	5-10%, predominantly T cells admixed with B cells	IgHV rearrangement not seen; no clonal B cell population	55% T cells; 25% B cells, polytypic surface immunoglobulin expression	“Diagnostic features of a lymphoproliferative disorder are not seen”	Harvard

Meanwhile, in 1993, at age 30, the patient began a self-directed health regimen. This included a near-vegetarian diet with occasional seafood, filtered water, over-the-counter nutritional supplements, and exercise (mainly resistance training with some aerobic activity consisting of power walking). He avoided processed foods and obtained organic or unsprayed produce whenever possible.

In May 2003, the patient, now aged 39, developed fever, malaise, night sweats, and fatigue. A Coombs positive hemolytic anemia secondary to chronic lymphocytic leukemia was diagnosed. A marrow examination showed 90% to 100% replacement of the marrow by lymphocytes. Treatment with steroids, rituximab, and cytotoxic drugs was recommended.

However, the patient declined the recommended therapy and instead amended his supplement regimen to include conjugated linoleic acid, whey protein with lactoferrin, and the botanical extract artemisinin. Despite feeling poor, he walked and swam daily. By October 2003, a few months after the episode began, the hemoglobin had risen to a normal value of 13.4 g/dL. The constitutional symptoms abated, and the patient made a complete clinical recovery.

In May 2006, when the patient was 42, a bone marrow examination showed lymphocytic replacement of 30%. This was decreased compared to the 2003 examination during the hemolytic anemia episode when lymphoid aggregates had replaced 90% to 100% of the marrow.

In December 2009, the white blood cell count rose to 17,500/µL, with a lymphocytosis of 63%. Hemoglobin was 13.6 g/dL, and platelets measured 293,000/µL. The patient, now age 46 and asymptomatic, consulted author KB.

Based on early-phase clinical trials showing the efficacy of epigallocatechin-3 gallate in chronic lymphocytic leukemia, author KB prescribed a supplement formula containing reishi mushroom (*Ganoderma lucidum*), chaga mushroom (*Inonotus obliquus*), and green tea (*Camellia sinensis*) [2]. This formulation was chosen because the green tea fraction provided approximately 1200 mg daily of epigallocatechin-3-gallate.

In addition to this directed anticancer treatment, the patient’s constitutional resistance to cancer progression was assessed via detailed testing of inflammation and glucose metabolism. High-normal fibrinogen levels and modest elevations in D-dimer and C-reactive protein were interpreted as signifying hypercoagulability and mild generalized inflammation. Although levels of insulin, blood glucose, and fructosamine were normal, C-peptide was above ideal at 5.3 ng/mL, suggestive of aberrant glucose metabolism. In addition, 25-hydroxy vitamin D was 46.7 ng/mL, slightly below the optimum range of 50 to 70 ng/mL.

To address these issues, the patient was placed on high-dose fish oil containing eicosapentaenoic acid (10.8 g) and docosahexaenoic acid (2.4 g daily), curcumin (4 g daily), vitamin D3, *Scutellaria baicalensis*, and probiotics. Calcium and magnesium were added to address a reported decrease in lumbar vertebral bone density.

Despite this approach, by October 2010, the white blood cell count had reached 47,800/µL, with lymphocytes at 81.6%. At this time, author BRK detected an immunoglobulin variable region heavy chain (IgVH) mutation in the peripheral blood by PCR.

In December 2010, author KB increased the dose of epigallocatechin-3-gallate to 4 g daily, basing the dose on that used in a clinical trial at Mayo Clinic [2].

In February 2011, two months after the patient began the higher dose of epigallocatechin-3-gallate, the white blood cell count peaked at 50,600/µL, with 84% lymphocytes. Fibrinogen, D-dimer, C-reactive protein, and C-peptide levels had dropped to low-optimal ranges. The vitamin D level had reached a supranormal level of 110 ng/dL, and the dose of supplemental vitamin D was decreased.

In March 2011, the white blood cell count had dropped, measuring 30,600/µL. By August 2011, the white blood cell count was 6800/µL, with a lymphocyte fraction of 49%.

In January 2012, when the patient was 48, a marrow examination at the Dana-Farber Cancer Institute demonstrated slightly elevated cellularity at 60%. However, lymphocytes represented only 5% to 10% of the cellularity. Flow cytometry of the aspirate also showed no evidence of a lymphoproliferative disorder. These findings were interpreted as being inconsistent with a lymphoproliferative disorder.

In September 2014, when the patient was 51, the marrow was hypercellular at 80%. However, lymphocytes again comprised only 5% to 10% of the cellular component. Of particular note, these lymphocytes were specifically tested and found to be predominantly CD3 positive T cells, whereas B cells would have been consistent with chronic lymphocytic leukemia. For a second time, flow cytometry of the aspirate showed no molecular evidence of a lymphoproliferative disorder. IgVH mutation was no longer detected on PCR. The entire study was interpreted as being without evidence of a lymphoproliferative disorder.

In 2015, more than three years after the documented remission, the patient remains well at age 52. He takes epigallocatechin-3-gallate (1200 mg) daily and maintains his self-directed lifestyle regimen.

## Discussion

We have here described two exceptional events in a man diagnosed at age 28 with chronic lymphocytic leukemia. The first event occurred at age 39: a remission of secondary autoimmune hemolytic anemia. To the authors’ knowledge, remission of secondary autoimmune hemolytic anemia without conventional treatment has never been reported in an adult. This outcome followed treatment with three nonprescription agents: 1) the lactone artemisinin, extracted from *Artemisia annua*; 2) conjugated linoleic acid; 3) whey protein with lactoferrin. All three have demonstrated anticancer activity in extensive preclinical testing [3,4].

The second exceptional event is the complete remission from chronic lymphocytic leukemia, achieved at age 48, documented by molecular studies, and related in time to supplemental curcumin, fish oil, vitamin D, *Scutellaria baicalensis*, and perhaps most importantly, epigallocatechin-3-gallate.

Several plausible mechanisms related to these supplements can be enumerated. In chronic lymphocytic leukemia patients, fish oil downregulates nuclear factor kappa-B (NFκB) activation in lymphocytes [5]. Higher vitamin D levels predict a longer time to first treatment in chronic lymphocytic leukemia [6]. Note that supplementation in this patient led to a supranormal vitamin D level in early 2011, shortly before remission. *Scutellaria baicalensis* causes apoptosis of myeloma cells in culture [7].

The timing of this second remission, shortly after an increase in dose of epigallocatechin-3-gallate to 4 g daily, is provocative, as is the concurrent use of curcumin. Preclinical studies show that epigallocatechin-3-gallate induces apoptosis in CLL B cells [2]. Whereas clinical trials have found epigallocatechin-3-gallate alone active against chronic lymphocytic leukemia, preclinical work suggests curcumin may potentiate the antitumor effect [2,8].

IgHV mutation, as seen in this case, is associated with a more indolent course in CLL [9]. Countering the argument for indolence in this case, however, is the patient’s initial presentation with lymphocytosis, splenomegaly, mild anemia, and thrombocytopenia. This presentation is consistent with Rai Stage III or IV, or Binet Stage C, of which all are unfavorable prognostic categories. Lastly, the malignant clone originally co-expressed CD5, a hallmark of classic chronic lymphocytic leukemia; the loss of CD5 expression that occurred, in this case, suggests that the patient’s lymphoproliferative disorder may have been an atypical form of chronic lymphocytic leukemia. What role, if any, the presence of IgHV mutation and early loss of CD5 expression may have played in the regression of this patient's CLL is unknown. Also unknown are other features of the tumor or host that may have helped sensitize the tumor to EGCG or may have contributed in some other way to the complete regression seen here. 

Standard treatment with anti-CD20 antibody and cytotoxic chemotherapy improves overall chronic lymphocytic leukemia survival, but is not curative. Novel therapies, including ibrutinib, idelalisib, and ABT-199 also improve survival, but curative potential is likely lacking. Prolonged therapy may be needed, which could be challenging, considering ibrutinib can cost $100,000 USD annually [9].

Hematopoietic stem cell transplant (HSCT) does offer a cure to some patients with chronic lymphocytic leukemia, with 45% remaining disease-free for five years. However, HSCT is appropriate only in high-risk chronic lymphocytic leukemia, because treatment-related mortality approaches 30% at two years. Additionally, chronic graft-versus-host disease decreases the quality of life in 25% of HSCT survivors [9]. Finally, costs related to HSCT typically exceed $80,000 USD in the first year after transplantation [10]. 

## Conclusions

Standard therapy for chronic lymphocytic leukemia fails to provide a cure. Novel targeted therapies are neither curative nor financially accessible for many patients. The safety risks of HSCT, the only conventional curative procedure, limit this option to a minority of patients. Therefore, the exceptional response of one chronic lymphocytic leukemia patient while taking epigallocatechin-3-gallate suggests that this botanical extract may offer a safe, inexpensive, and effective treatment for some patients, and that it should be vigorously investigated.
